# Scientific Publications on Nursing for COVID-19 in Patients With Cancer: Scoping Review

**DOI:** 10.2196/39012

**Published:** 2022-11-25

**Authors:** Vivian Cristina Gama Souza Lima, Raquel de Souza Soares, Willian Alves dos Santos, Paulo Alves, Patricia dos Santos Claro Fuly

**Affiliations:** 1 Academic Program in Health Care Sciences Fluminense Federal University Niterói - RJ Brazil; 2 National Cancer Institute Rio de Janeiro Brazil; 3 Interdisciplinary Health Research Centre, Institute of Health Sciences Catholic University of Portugal Porto Portugal

**Keywords:** COVID-19, review, nursing, coronavirus infection, oncology nursing

## Abstract

**Background:**

The needs of patients with cancer must be met, especially in times of crisis. The advent of the pandemic triggered a series of strategic actions by the nursing team to preserve the health of patients and professionals—hence the importance of studies on nursing care actions provided to patients with cancer during the COVID-19 pandemic. It is known that these patients are susceptible to severe COVID-19. However, no previous review has summarized the findings of scientific studies on nursing for COVID-19 in patients with cancer.

**Objective:**

This study aims to map the topics addressed in scientific studies on nursing for COVID-19 in patients with cancer.

**Methods:**

A scoping review was conducted using the methodology described in the *Joanna Briggs Institute Reviewers' Manual 2015*. The research question was elaborated using the population, concept, and context framework: What topics have been studied in nursing publications about COVID-19 in adult patients with cancer? The searches were carried out in 8 databases between April and November 2021 without time restrictions.

**Results:**

In total, 973 publications were identified using the search strategies in the databases, and 12 papers were retrieved by consulting the references. A total of 31 (3.2%) publications were included in the final analysis, generating 4 thematic categories on the subject: “restructuring the services: how oncology nursing was adapted during the pandemic,” “experiences of patients and performance of the nursing team during the COVID-19 pandemic,” “protocols and recommendations for dealing with the COVID-19 pandemic,” and “challenges and the role of oncology nurses facing the COVID-19 pandemic.”

**Conclusions:**

Several strategies used by oncology nurses to face the COVID-19 pandemic in the international scenario were identified. Reports about the restructuring of services and the team's reactions to the pandemic predominated. However, there is a lack of reports regarding emotional support strategies for health care professionals. Another gap identified was the scarcity of clinical studies on the activities developed by oncology nurses. Therefore, there is a need for clinical research in the oncology area and emotional coping strategies to support oncology nurses.

## Introduction

Cancer is 1 of the main public health issues in the world, especially in developing countries, and it is already among the 4 main causes of death before the age of 70 years in most countries. The incidence and mortality from cancer have been increasing considerably, whether due to aging, population growth, or the change in the distribution and prevalence of risk factors, especially those associated with socioeconomic development [1].

In this context, it is noteworthy that patients with cancer are susceptible to developing infections. Despite the benefits of cancer treatment, it may cause the expansion of immunosuppression, making these patients more vulnerable to bacterial, fungal, and viral infections [2].

The world has been affected by the outbreak of COVID-19, a public health emergency. Since the emergence of SARS-CoV-2 in late 2019 in China, the worldwide spread has been rapid, with over 395 million confirmed cases and over 5.7 million deaths reported worldwide as of January 2022 [3-6]. It is observed that infected patients can present different manifestations and results, especially when considering the oncological disease. Therefore, it is essential to identify, through studies, the main characteristics of those infected in order to help allocate the right resources and improve the quality of care [7].

The SARS-CoV-2 infection has a mild course in most people, but in a significant portion of the population, the condition progresses to a severe respiratory disease characterized by hyperinflammatory syndrome, multiple-organ dysfunction, and death [8]. This is because some patient subgroups, such as the elderly and individuals with chronic conditions, such as hypertension, diabetes, and chronic lung diseases, have been shown to have an increased risk of morbidity and mortality when affected by COVID-19 [9].

Literature data indicate that patients with cancer undergoing active treatment are at greater risk of developing serious events related to COVID-19, requiring admission to an intensive care unit [10]. There are reports of early studies from China that demonstrated a 2- to 4-fold increase in COVID-19 mortality among patients with cancer compared to those without cancer, while other smaller studies reported a 29% case fatality rate and worse outcomes among patients with cancer infected with COVID-19 [9].

In this way, it is understood that the needs of patients with cancer must be met, especially in times of crisis. The advent of the pandemic triggered a series of strategic actions by the nursing team to preserve the health of patients and the team—hence the importance of studies to guide the nursing care provided to patients with cancer in the context of the COVID-19 pandemic [11].

The role of nursing has undergone significant changes, mainly due to the need for changes in the nursing education process. Currently, nurses are increasingly trained to promote and improve the quality of clinical practice and provide support in patient and community care in all aspects. With the pandemic, new opportunities and threats have emerged due to the introduction of new technologies in the health area, which requires the nursing team to develop new digital skills [12].

It is noteworthy that the nursing team is of fundamental importance in patient care in the face of the pandemic. Within health services, nurses are the main direct caregivers providing vital services and, in this way, are considered the system's backbone. It is a fact that the pandemic has shown that many health environments are also workplaces where these professionals face high risks of occupational exposure to physical and mental illnesses [13]. Instead of absenteeism, during the pandemic, an increase in presenteeism—the problem of workers being on the job but, because of illness (in this case COVID-19), not fully functioning—has been observed in a hospital in Australia. Frontline nurses were more affected by SARS-CoV-2 than all other health care professions [13].

To find the publications on the subject, a preliminary search was carried out in the International Prospective Registry of Systematic Reviews (PROSPERO), Cochrane Systematic Review, and Medical Literature Analysis and Retrieval System Online (MEDLINE). We searched the literature for protocols and reviews on the topic and identified 30 studies by using the descriptors “COVID-19,” “nursing,” and “cancer” connected by the Boolean operator AND. Only 2 studies related to the topic were found, a review about home care and palliative care [14] and a research protocol published as a conference proceeding [15], indicating the existence of a gap in the literature. Such limitations may compromise the understanding of actions aimed at nursing care for patients with cancer and COVID-19.

Thus, it is recognized that a broad literature review can bring new knowledge to the subject. Therefore, this scoping review's objective was to map the topics addressed in scientific publications on nursing for COVID-19 in patients with cancer.

## Methods

### Design

A scoping review was carried out using the Joanna Briggs Institute's (JBI) [16] method that provides an overview of the evidence, being recommended to authors who intend to answer specific research questions about the nature and diversity of evidence on a given topic or identify existing gaps [17]. The following 5 steps were completed: selection of the research question, identification of relevant publications, study selection, data extraction, and data synthesis and discussion of findings [17-19]. The protocol for this scoping review has been registered with the Open Science Framework [20].

### Selecting the Research Question

The research question was created using the Population, Concept, and Context (PCC) framework as follows:

- Population: adult patients (defined by the World Health Organization [WHO] as 18 years or over) with any type of cancer
- Concept: nursing care in the different scenarios of the nursing activity
- Context: the COVID-19 pandemic

Therefore, the following research question was explored: What topics have been studied in nursing publications on COVID-19 in adult patients with cancer?

### Identifying Relevant Publications

The following databases were used to retrieve publications: the Cumulative Index to Nursing and Allied Health Literature (CINAHL), the National Center for Biotechnology Information (NCBI/PubMed), the Latin American and Caribbean Health Sciences Literature (LILACS), Scopus, and the Excerpta Medica Database (Embase). The gray literature was searched for additional relevant publications using Mednar and the *Gray Literature Report*. Subsequently, the search was extended to the Virtual Health Library (VHL) and the Brazilian Digital Library of Theses and Dissertations (BDTD, in Portuguese). The search was restricted to studies published from March 11, 2020, to date, considering the date on which the pandemic state was declared [5]. No language restrictions were used.

The descriptors “coronavirus infections,” “oncology,” and “nursing” were combined using the Boolean operators AND and OR to construct the search strategy, which was defined considering the specificities of each database.

Primary and secondary studies were included regardless of their methodological approach. Therefore, original papers, literature reviews, term papers, theses, and dissertations were eligible if they were available for free or through subscriptions made by the authors' institution. The following exclusion criteria were used: duplicates, errata, commentary papers, research protocols, booklets, studies not addressing the research question, and papers not available in full text.

### Study Selection

The study selection was carried out from April to November 2021. After the search strategy was used in the databases, the results were exported to EndNote, through which the papers were grouped, and duplicates were removed before title and abstract screening. Two different reviewers conducted this step guided by pre-established inclusion criteria and the research question to systematize the review and reduce the risk of bias. Disagreements between the reviewers were resolved through discussion with a third reviewer. The selection process is depicted in a Preferred Reporting Items for Systematic Reviews and Meta-Analyses (PRISMA) flowchart [16].

### Data Extraction

The data extraction was developed using a form created based on the review's objective and research question, with the following variables: bibliographic information (origin, type of publication, title, authors, descriptors, year, country, and language), objectives, study design, methodological approach (data collection, period, location, analytical method, and treatment of data), conclusions, and recommendations. The authors pilot-tested the form before the data extraction, making the necessary adjustments.

Two steps were conducted to reach the final sample. First, the titles and abstracts of the publications were screened based on the eligibility criteria. In the second round of screening, the full texts were read to ensure their congruence with the research question and 2 independent reviewers validated the process. After these steps, the retrieved publications were listed and numbered according to the chronological order of the data collection and sorted in an electronic spreadsheet.

### Data Synthesis and Discussion of Findings

The studies were synthesized using a chart with the main characteristics and points of interest of each study. A figure was created to synthesize information about the studies' countries, years of publication, and designs. A discussion of findings was conducted using descriptive statistics and narrative synthesis.

## Results

### Identifying Relevant Publications

**Figure 1:**
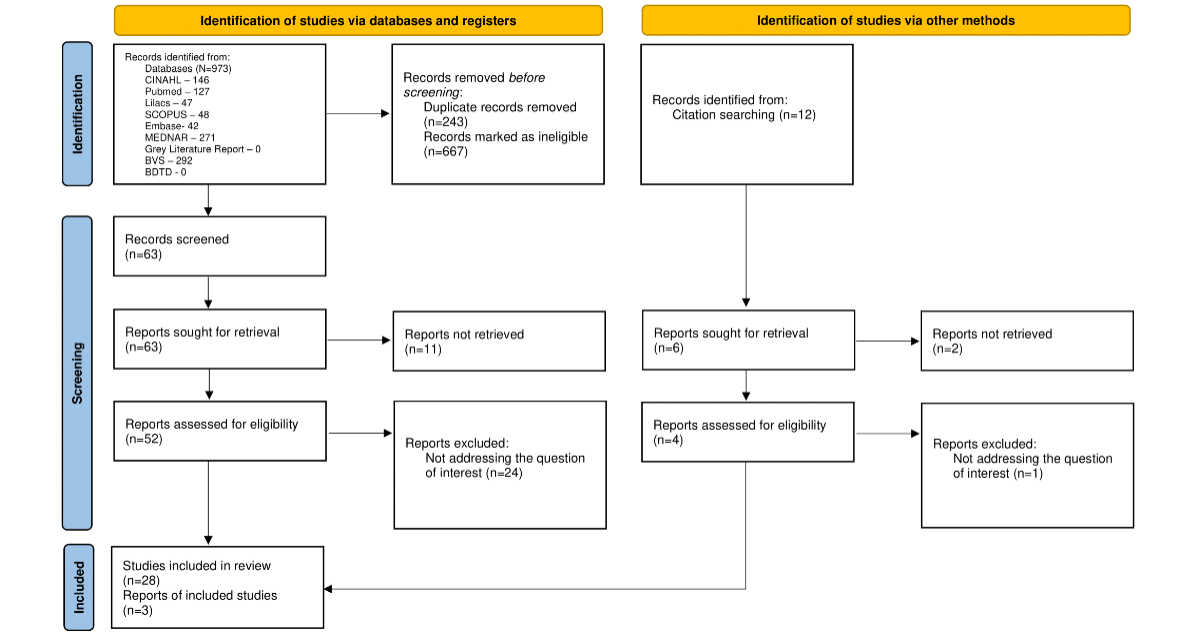
Preferred Reporting Items for Systematic Reviews and Meta-Analyses (PRISMA) flowchart of study selection.

The search strategies allowed us to identify 973 records, from which 243 (25%) duplicates were removed. After the title and abstract screening, 63 (6.5%) documents remained and were read in full. After applying the exclusion criteria, 29 (46%) documents remained. After reading the references of these documents, 12 additional records were identified, of which 3 were selected, resulting in 31 publications, as shown in Figure 1.

### Selection of Studies

**Figure 2:**
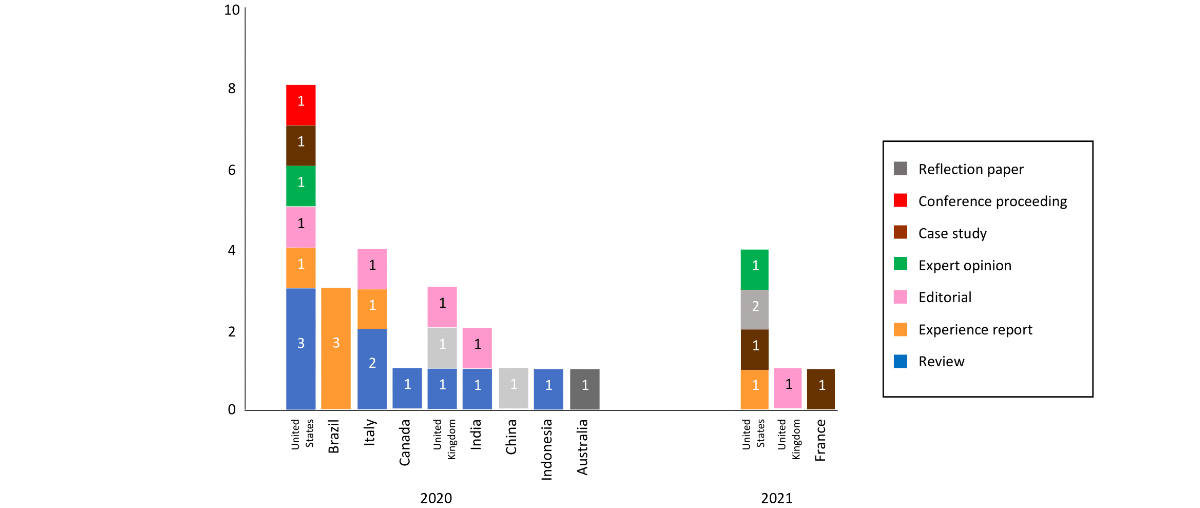
Distribution of papers by year of publication, country, and type (N=31).

The main characteristics of the included studies are presented in Tables 1 and 2 and Figure 2. A narrative summary was prepared using categories that emerged from the results found, and the JBI Appraisal Tool was adopted to assess the methodological quality and risk of bias.

**Table 1:** Synthesis of the characteristics of the included studies (N=31).

Study ID	Title	Journal	Authors	Level of evidence
S1	A COVID-19 Screening Tool for Oncology Telephone Triage	*Supportive Care in Cancer*	Elkin et al [21]	4a
S2	A New Proactive Virtual Resource Center Navigation Model Identifies Patient Risk Factors to Reduce Barriers to Cancer Care During the COVID-19 Pandemic	*Supportive Care in Cancer*	Bigelow et al [22]	4b
S3	The Role of Patient Safety Service in the Fight Against COVID-19 in a Hospital	*Enfermagem em Foco*	Cardoso et al [23]	5c
S4	First Case of COVID-19 in an Oncological Palliative Care Unit: Experience Report	*Enfermagem em Foco*	Santiago et al [24]	5c
S5	Defining Moments: A Nurse’s Touch	*Heath Communication*	Baglia [25]	5c
S6	Comment on “Pathways to Psychological Wellbeing for Patients With Bladder Cancer and Their Partners-in-Care” and Contextualization in the COVID-19 Pandemic	*European Journal of Oncology Nursing*	Caruso et al [26]	5c
S7	COVID-19 and Cancer Nursing: Challenges and Opportunities	*Canadian Oncology Nursing Journal*	Booker [27]	5c
S8	COVID-19’s Implications for People With Cancer and Oncology Nurses Oncology Nurses	*News and News - ONS^a^ Voice*	Sheldon [28]	5c
S9	Editorial: The Critical Role of Nurse Practitioners in the Care of Cancer Patients	*European Journal of Cancer Care*	Weller [29]	5c
S10	Infusion of Antineoplastic therapies in the Home	*Oncology Nursing Forum*	ONS [30]	5c
S11	Managing the Journey of Patients Under Chemotherapy in a Pandemic Era: A Nursing Perspective	*Chemotherapy*	Gualandi et al [31]	4a
S12	Mitigating Strategies and Nursing Response for Cancer Care Management During the COVID-19 Pandemic: An Italian Experience	*International Council of Nurses*	Zeneli et al [32]	4a
S13	Nursing Navigation in Breast Cancer Care During the Pandemic: An Experience Report	*Journal of Nursing and Health*	Osorio et al [33]	5c
S14	Navigating a Global Pandemic: How Nurses in Florida Responded	*The Florida Nurse*	Marshall [34]	4a
S15	Nurses' Role in Providing Comprehensive Communication, Prognostication, and Palliative Care During the COVID-19 Pandemic	*Journal of Hospice & Palliative Nursing*	Koch et al [35]	4d
S16	Oncology Nursing Challenges During COVID‐19 Outbreak: Precautions and Guidance	*Asia‐Pacific Journal of Oncology Nursing*	Shankar et al [36]	5c
S17	Oncology Nursing During a Pandemic: Critical Reflections in the Context of COVID-19	*Seminars in Oncology Nursing*	Paterson et al [37]	4a
S18	Oncology Nursing Workforce: Challenges, Solutions, and Future Strategies	*The Lancet Oncology*	Challinor et al [38]	4a
S19	Palliative Care Challenges and Strategies for the Management Amid COVID-19 Pandemic in India: Perspectives of Palliative Care Nurses, Cancer Patients, and Caregivers	*Indian Journal of Palliative Care*	Pai et al [39]	5c
S20	Patient Satisfaction With Nurse-Led End of Treatment Telephone Consultation for Breast Cancer During COVID-19 Pandemic	*The Breast Journal*	Schuster-Bruce et al [40]	5c
S21	Nurse Navigators’ Telemonitoring for Cancer Patients With COVID-19: A French Case Study	*Supportive Care in Cancer*	Ferrua et al [41]	4d
S22	Primary Palliative Care Clinical Implications: Oncology Nursing During the COVID-19 Pandemic	*Clinical Journal of Oncology Nursing*	Rosa et al [42]	4d
S23	The Psychological Pressures of Breast Cancer Patients During the COVID-19 Outbreak in China: A Comparison With Frontline Female Nurses	*Frontiers in Psychiatry*	Cui et al [43]	4a
S24	The Role of the Oncology Nurse Navigator in Establishing an Epic Workflow for Virtual Multidisciplinary Clinics During Covid Restrictions	*LVHN Scholarly Works*	Beaupre et al [44]	5c
S25	COVID-19 and Cancer Care	*The British Journal of Nursing*	Foulkes [45]	5c
S26	Sarah Cannon Virtual Breast Cancer Support Group	*Journal of Oncology Navigation & Survivorship*	Sarah Cannon [46]	5c
S27	Colorectal Cancer Surgery in the Coronavirus (COVID-19) Pandemic	*Gastrointestinal Nursing*	Taylor et al [47]	5c
S28	Fighting Cancer in Coronavirus Disease Era: Organization of Work in Medical Oncology Departments in Emilia Romagna Region of Italy	*Future Oncology*	Brandes et al [48]	5c
S29	The Cancer Nurse as Primary Palliative Care Agent During COVID-19	*Cancer Nursing*	Rosa et al [49]	5c
S30	Nurse’s Roles in Protecting Cancer Patients During COVID-19 Pandemic	*Jurnal Bedah Nasional*	Nuryani et al [50]	5c
S31	Navigating the COVID-19 Pandemic as an Oncology Nurse	*Oncology Times*	Nalley [51]	5c

^a^ONS: Oncology Nursing Society.

**Table 2:** Synthesis of the topics of the included studies (N=31).

Study ID	Subject
S1	Development and implementation of a COVID-19–screening tool for oncology telephone service. The tool was developed and implemented in clinical practice, facilitating patient triage and patient tracking in various outpatient settings.
S2	Implementation, associated interventions, and results of the proactive navigation model of a virtual resource center. Successfully transitioned to a new proactive, virtual outreach program to educate, advocate, resource, and support patients with cancer during the COVID-19 pandemic.
S3	Experience of the Patient Safety Center, in the face of COVID-19, in a hospital unit. The lived experience shows the importance of the Patient Safety Center, which aims to promote a safe health service.
S4	Report on nursing care to the first patient in oncological palliative care with COVID-19 in a hospital in Rio de Janeiro. The rapid worsening of the disease, isolation, absence of a caregiver/family member, and risk of contamination of the team in the context of the pandemic made nursing care more specific and careful.
S5	Report on the relationship between a patient, the nurse, and the physician who accompanied the patient with lymphoma during treatment and differences between the moments before and during the COVID-19 pandemic.
S6	Continuous psychological assessment of patients with cancer, especially in unusual health conditions, such as COVID-19, can improve nursing practice and patient outcomes.
S7	Discuss the role of nurses in disseminating information during the pandemic.
S8	Implications of the COVID-19 pandemic for oncology nurses and patients with cancer.
S9	Performance of nurse-led clinics in supporting patients with cancer during the COVID-19 pandemic. The services led by nurses showed good performance and quality, and the teleservice performed by nurses indicated a high level of patient satisfaction.
S10	Guidelines to be followed by any health care organization offering systemic anticancer therapy at home, established by the ONS^a^ to avoid interruption of patient care.
S11	Update on the needs, experiences, and responses to treating patients treated with chemotherapy in a context of high risk of contagion due to the COVID-19 pandemic. New solutions to emerging problems are implemented, even without scientific evidence.
S12	Description of a cancer center's COVID-19 emergency response to allow other nursing organizations to determine what elements might help manage an increase in patients in their own environment.
S13	Navigator nurses' role in assisting patients with breast cancer during the new coronavirus pandemic in a private hospital in Southern Brazil.
S14	Florida nurses' response to the COVID-19 pandemic: changes in academia and research during the pandemic, the negative consequences of COVID-19 on cancer care, and the innovative model created by perioperative services to care for patients with COVID-19.
S15	Case report of assistance to the mother of a child with end-stage cancer with whom providers had not discussed care goals and prognosis during the COVID-19 pandemic.
S16	Challenges for oncology nursing during the COVID-19 outbreak: supporting patients with cancer during treatment, managing case care amid the COVID-19 crisis, and assessing the risk of exposure to coronavirus infection in the face of cancer treatment to avoid anxiety and panic among patients with cancer.
S17	Critical reflection on COVID-19 in the context of oncology nursing with recommendations for caring for people affected by cancer during this pandemic. Nurses participate in the development and implementation of policies regarding standards of care and play a key role in the management of COVID-19 in the year marked as the International Year of Nursing.
S18	A narrative review on challenges faced by oncology nurses during the COVID-19 pandemic, including shortages of nurses and specialized staff, occupational safety and burnout concerns, and possible solutions to address these challenges.
S19	Palliative care challenges and strategies for management during the COVID-19 pandemic in India.
S20	Benefits of teleconsultation carried out by nurses to patients with breast cancer during the COVID-19 pandemic.
S21	The use of a new application effectively monitored patients with cancer and reduced contact with other people.
S22	Integrating palliative care into the practice of oncology nursing helps health organizations and cancer centers be better equipped to meet the holistic needs of patients with cancer and their families.
S23	Psychological status of patients with breast cancer and female nurses at the peak of the COVID-19 outbreak.
S24	Safe options for conducting multidisciplinary clinics remotely, maintaining the same level of quality, during a time when at least half of providers and browsers were working remotely during COVID-19 restrictions.
S25	Challenges for oncology nurses in treating patients with cancer facing the COVID-19 pandemic.
S26	Experience of a virtual group with women with breast cancer in the face of the COVID-19 pandemic.
S27	Guidance for specialist nurses to help patients decide whether to proceed with or delay colorectal cancer surgery during the COVID-19 pandemic, guide them along new paths, address their concerns, and provide preoperative assessment and support.
S28	Various topics about the current pandemic. COVID-19 will continue to disrupt society and cancer care in 2021, but the arrival of vaccines brings hope for a decrease in serious infections.
S29	Measures applied to reduce the spread of COVID-19 in clinical oncology departments.
S30	The role of oncology nurses in strategic roles during the pandemic includes educating the patient's family, coordinating with other health teams, triaging patients by phone, and caring for their own mental health.
S31	Challenges for health professionals and oncology nurses in coping with the pandemic: protocols for chemotherapy administration, patient protection, and guarantee of PPE^b^ to health professionals.

^a^ONS: Oncology Nursing Society.

^b^PPE: personal protective equipment.

### Data Extraction

All publications were retrieved from databases or citations search, and no documents were selected from the gray literature. Regarding the language and type of publication, 28 (90%) of the 31 studies were written in English and most were published in scientific journals (n=28, 90%). With regard to research design, 9 (29%) were literature reviews, 6 (19%) experience reports, 6 (19%) editorials, only 3 (10%) original papers, 3 (10%) case studies, and 1 (3%) publication in a conference proceeding. Almost half of the studies were developed in the United States, with a total of 13 (42%) publications, followed by Italy with 4 (13%) and the United Kingdom also with 4 (13%) publications. Of the 31 publications, 25 (80%) were published in 2020.

### Data Synthesis and Discussion of Findings

**Figure 3:**
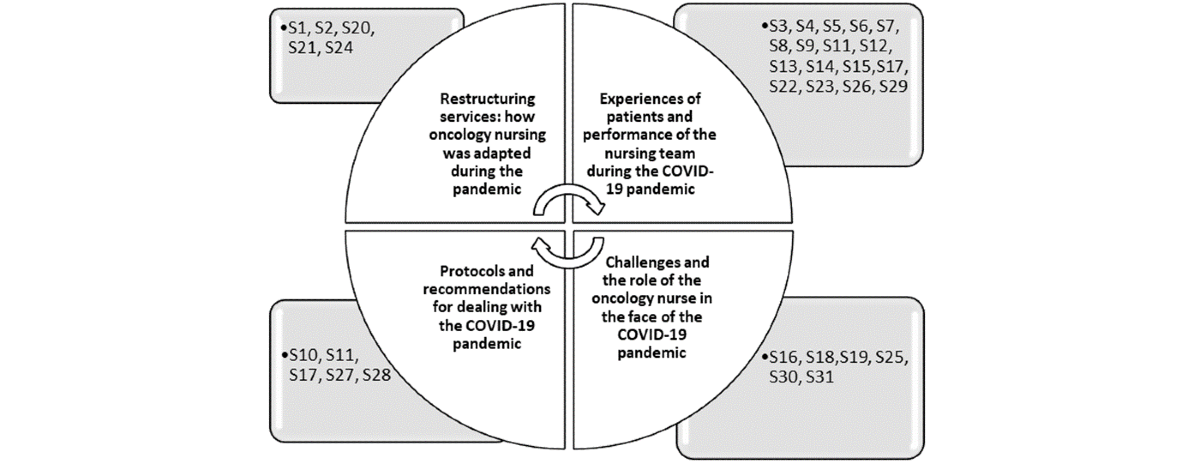
Distribution of papers by thematic category (N=31).

From the analysis of the papers, 4 categories emerged, as shown in Figure 3.

#### Restructuring the Services: How Oncology Nursing Was Adapted During the Pandemic

This category includes how certain services have been reinvented to provide quality care remotely, given the need for social distancing during the pandemic. Of the 31 studies, 2 (6%) addressed nursing care through telephone monitoring and 2 (6%) others outlined different strategies for remote care. The development and implementation of a telephone tool included questions about patients' cancer history and current treatments and an emphasis on asking patients about their symptoms (which may be related to COVID-19 or cancer and treatment) [19]. In this way, nurses made informed decisions and assertive triage. Similarly, a tertiary cancer center in Paris, France, rapidly reorganized cancer treatment pathways to protect patients with cancer from severe COVID-19 and ensure that these patients continue receiving optimal cancer care [41].

To deal with the increasing barriers, another specialized oncology center has adopted a new navigation strategy based on virtual resources targeting the needs of the most vulnerable patients with cancer [22]. The model, led by nurse navigators, is patient centered and prioritizes visits carried out by telephone or video. With the partial closure of the service in March 2020, the previous navigation model could not meet the needs of patients with cancer and their families. Therefore, the decision to restructure this virtual model was efficient and effective. The navigation model provided support and improved care and safety for patients and family members who could not get these services during the pandemic [22].

In another paper, the navigation team of the Lehigh Valley Health Network [44] needed to create safe options to carry out its multidisciplinary consultations remotely, maintaining the same level of quality during a period in which at least half of the providers were working remotely [44]. Due to the pandemic, the demand for virtual clinical experiences required several clinical and administrative tasks from nurses. Despite the restrictions, patients and providers have appreciated the opportunity to safely continue their multidisciplinary efforts, which remains an option for off-site patients and providers.

The authors of another study evaluated the benefits of teleconsultation during COVID-19 and found that although some patients were satisfied with this approach, at least half of the sample still preferred face-to-face appointments [40].

#### Experiences of Patients and Performance of the Nursing Team During the COVID-19 Pandemic

An experience report was identified about the experiences lived by a patient during the pandemic. The patient mentions noticeable differences between the pre- and postpandemic periods in the appointments with nurses and physicians, emphasizing changes in social behaviors, such as lack of hugs or handshakes and the need to have the face constantly hidden behind a mask [25].

Studies examining the experiences lived by nurses were also found, discussing patient safety issues in the hospital scenario [23], the management of the nursing team, and the challenges that arose along with the first case of COVID-19 in a palliative care setting [24], the implementation of a remote approach to monitor and assist patients with breast cancer in palliative care [33], and the experience of carrying out a virtual group for women with breast cancer in the face of the pandemic [46].

Another way of describing current experiences is through case studies and the publication of narrative reviews, as in a paper that discusses the benefits of psychological therapies for patients with cancer, increasing their well-being and favoring the performance of the nursing team [26]. A study in China showed that patients with breast cancer had better psychological adaptation than frontline nurses at the COVID-19 epicenter in Wuhan [43]. Other authors discuss the different roles and attributions given to nurses during the pandemic: dissemination of information, service leadership, and telehealth, all with a high level of responsibility and expectations, being extremely relevant in the current scenario [27-29]. In addition, 2 (6%) papers describe the “nursing response to COVID-19” and what would be the reaction of this team to the pandemic in the context of nursing work. The intention is to allow other nursing organizations, based on these reports, to determine which elements may be useful to manage the increased number of patients in nursing care settings. The top topics covered changes in academia and research during the pandemic, the negative consequences of COVID-19 on cancer care, and the innovative models created by health care institutions [32,34].

With regard to palliative care, nurses must be trained to deal with these cases during the pandemic. A case study [35] reports the case of a mother of a child with end-stage cancer with whom the team had not yet discussed the prognosis. The authors conclude that the early introduction of palliative care and greater efforts to train and encourage conversations about palliative care by nurses can improve the quality of life for these patients and their families. Another paper addresses topics aimed at training clinical oncology nurses working in all settings with primary palliative care skills during the COVID-19 pandemic [42].

In addition to these topics, other diverse topics are addressed through newsletters and papers, such as vaccines, death and contagion statistics, and measures to reduce the spread of COVID-19 in hospital and home environments [28,48]. It is worth mentioning that these publications were extremely important throughout the pandemic, as new protocols and recommendations were still being created.

#### Protocols and Recommendations for Dealing With the COVID-19 Pandemic

Of the 31 publications, 1 (3%) cites that to avoid interrupting the treatment of patients, the Oncology Nursing Society (ONS) established guidelines to be followed by any health organization providing systemic anticancer therapy

at home [30]. These guidelines include assessment of the complexity and risk of complications, assessment of the home environment and patient and family readiness to receive care at home, handling of antineoplastic agents, the correct disposal of waste, and indications concerning personal protective equipment (PPE), among other recommendations. In addition, the study emphasizes the need for informed consent from the patient, the need for keeping a spill kit at home, and the prerequisites that nurses must meet to administer anticancer drugs at home.

Furthermore, 1 (3%) of the studies reinforces the importance of reviewing and following institutional recommendations from specialized, state, and governmental agencies [37]. The paper presents recommendations for identifying and tracking contacts, guidelines for using PPE according to WHO recommendations, and an algorithm that can be used to ensure core staff availability for specialty areas prior to the deployment of staff to a labor pool. Nurses are advised on whether to proceed or postpone colorectal cancer surgery during the pandemic and what issues should be addressed during preoperative cancer patient care [47]. It is noted that the National Health Service (NHS) Action Plan reinforces that national health services and local authorities must implement plans to ensure that people receive essential care and support during all phases of a future pandemic through educational materials to educate patients about the surgical treatment of cancer and prioritizing patients according to their severity.

#### Challenges and the Role of Oncology Nurses Facing the COVID-19 Pandemic

WHO declared 2021 the International Year of Nursing, recognizing the tremendous work carried out by these workers. In the same year, the world was hit by the pandemic, the great challenge for oncology nurses was delivering care during the COVID-19 pandemic and not getting sick [36]. Although oncology nurses routinely use PPE, shortages of this type of material have been reported in many countries. In addition, healthy people are at risk of spreading the infection. Therefore, maintaining a balance between exposure to the virus and delivery of cancer treatment should be a priority for nurses to avoid unnecessary anxiety and panic.

A worldwide oncology nursing workforce is critical to achieving the United Nations 2030 Agenda for Sustainable Development Goals: 3.4 (reduce premature mortality from noncommunicable diseases by one-third) and 3.8 (achieve universal health coverage) [38]. Unfortunately, challenges for a robust oncology nursing workforce include shortages, recruitment barriers, and burnout. The long-term effect of COVID-19 on cancer care worldwide is unknown, but the immediate interruptions of therapy, workforce consequences, and threats to standard cancer nursing practice are addressed in this paper.

The challenges faced by palliative care nurses have also been addressed. An Indian paper [39] reports the main causes of stress and suffering experienced by nurses during the COVID-19 outbreak. The fear of contracting the infection was the main concern that most nurses faced in direct contact with patients. In palliative care centers, nurses were anxious and concerned about the lack of staff, distancing from family members, and feeling alone in delivering care to dying patients and recognized other factors contributing to stress. For example, violence against COVID-19 heroes, such as physicians and other health care workers, has been reported in India, making the entire process of treating the COVID-19 pandemic infection more traumatic than ever.

The pandemic has made patients reluctant to seek help from health care services, reducing urgent referrals. Undoubtedly, this is due to a combination of the intention to avoid increasing the health care system's workload and the fear of contracting coronavirus. Another challenge mentioned in 1 (3%) of the studies reviewed [45] is the necessity to meet an influx of people with cancer in hospitals that also treat patients with COVID-19. Therefore, a consequence of the pandemic was the need to discuss risks that have never been addressed.

Health care workers are used to providing treatments to patients with advanced diseases, considering their quality of life and managing the associated risks. In the context of COVID-19, this was quickly rephrased. Treatments that expose patients to a greater risk of contracting the virus may no longer be viable in terms of outcomes and workload for services. Consequently, many treatments may not be initiated or are likely to be discontinued.

An open discussion of risks with patients is necessary and desirable, but they are difficult and inevitably lead to anguish, worry, and uncertainties. Authors report the need to associate the current challenges of this pandemic with the care of patients with cancer undergoing chemotherapy [31]. Another study [37] critically reflects the challenges faced in the pandemic, gives recommendations to the nursing team, and states that nurses are key stakeholders in developing and implementing policies regarding standards of care during the COVID-19 pandemic. This statement speaks a lot about the role of nurses in the current context. The different roles assumed by nurses in the pandemic complement each other. The complexities of COVID-19 revealed the importance of oncology nurses assuming their role as agents of primary palliative care in defense of the patient and protecting patients with cancer during the pandemic, with a focus on preventing transmission and providing support to patients and families [49-51].

## Discussion

### Principal Findings

The main issues addressed in the publications were the adaptation and restructuring of services. Several services reinvented themselves to provide quality assistance even remotely, as there was a need for social distancing during the pandemic. The focus of the service was telehealth, characterized by virtual consultations by video calls or by telephone and other remote approaches. The restructuring of the health service is a complex element, as it involves knowledge about where cases and deaths are concentrated [52]. It was not evidenced in this review, for example, the emergence of new positions or jobs or the duration of these adaptations. In addition, the emergence of clusters of COVID-19 cases and the dynamics of occurrence of these cases must be monitored, in addition to trend analysis, so that timely interventions or new adjustments are carried out.

Nurse navigation services were also adapted to the virtual model. Due to the high barriers to care during the pandemic, a new navigation strategy based on virtual resources has been adopted, targeting the needs of the most vulnerable patients with cancer. Navigator nurses generally lead the process, and most visits are done by telephone or video. Multidisciplinary teams have been challenged to carry out their approaches remotely, while keeping the same quality of service in a period when at least half of the providers and navigators were working remotely. It is concluded that adapting to a virtual model was efficient and effective, providing support and improved care for patients and family members who might not have received these services during the pandemic. The remote care model is characterized by devices, services, and interventions designed around the health and well-being needs of the patient, and related data are shared so that the patient can receive care as proactively and efficiently as possible [53].

The evidence shows that nursing teleconsultation has many benefits, and many patients were satisfied with this assistance modality during the COVID-19 pandemic. However, according to the findings of this research, a relevant part of the patients approached in some studies prefer face-to-face encounters. It was not evidenced in the findings that the teleservice has made the day-to-day activities of those involved difficult. Health professionals, patients, and their families were well adapted to the new reality, and this modality is considered a facilitator of assistance during the pandemic.

The experience reports revealed several aspects experienced in the pandemic scenario. The nursing team reports show experiences regarding the team's performance concerning patient safety issues in the inpatient and outpatient settings. Likewise, the team's management had to be adapted in the face of cases of COVID-19, as well as the role of nurse navigators and palliative care nurses who were required to conduct virtual approaches. Case studies and narrative reviews are useful to disseminate reflections and experiences via a singular and, simultaneously, plural history since they allow the creation of links between meanings and experiences lived in a given sociohistorical context [54].

The importance of support from the psychology service to patients with cancer and professionals facing important emotional problems during the pandemic is also highlighted. Nurses, seen as “heroes and heroines” in this context, needed to assume multiple roles and deal with new attributions and those notably known, such as disseminating information, carrying out leadership roles, and conducting telehealth encounters, all with responsibility and accountability. However, the reactions of this team of professionals are worrisome.

A study published in China [55] shows that health care workers have faced psychological consequences, such as anxiety, stress, and depression, since the beginning of the COVID-19 pandemic. Psychological impacts generated by the pandemic, already intense in the general population, are amplified in health professionals, especially those on the front line of care. In addition to being exposed to the virus, the nursing team is exposed to the lack of PPE and hospital supplies and the role of deciding which patients will receive further treatment. The author also states that after the outbreak of SARS, Japanese professionals started to consume more alcohol and tobacco due to the stress suffered, increasing posttraumatic stress. It is important to acknowledge that health care workers are 1 of the most vulnerable groups to burnout syndrome. This psychosocial phenomenon emerges as a response to chronic stressors present at work, characterized by emotional exhaustion, depersonalization, and reduced personal fulfillment at work.

Several recommendations and protocols were made to guide health teams and the population during the COVID-19 pandemic. The ONS has established guidelines to be followed by health care organizations that provide anticancer treatment at home. It is known that the health workforce was extremely demanded during the pandemic, with a large volume of work, changes in the workflow, and technological innovation. During the fight against COVID-19, health managers had to deal with several adversities, such as shortages of supplies, exhaustion of frontline professionals, technology implementations, and internal process changes [56].

During the pandemic, nurses needed to follow institutional recommendations. Several guidelines concerning the identification and tracking of contacts, the use of PPE, the organization of the workforce, and the allocation of personnel have been disseminated. In addition, specialist nurses were assigned roles such as assisting patients in decision-making regarding cancer treatment, addressing their concerns, and providing preoperative assessment and support [47].

Oncology nurses have faced major challenges in the COVID-19 pandemic. These challenges include managing patients with cancer, while preventing the risk of disseminating the coronavirus infection in a shortage of PPE and resources, which occurred worldwide. In this context, a study [57] states that nurses routinely face precariousness in the workplace and numerous problems in the health system, such as lack of infrastructure, scarcity of supplies, inadequate staffing, lack of PPE, work overload, low wages, and lack of training. All these factors contribute to the illness and stress of health care workers [57].

One of the groups most impacted by COVID-19 can be considered to be health professionals, as they have experienced measurable negative psychological impacts [58]. Nurses and multidisciplinary staff may have experienced a variety of stressors related to interruptions in routine work tasks, limited knowledge and data about the illness, and job security concerns. The various changes forced the team to learn and adopt new work tools quickly, and this unexpected need can cause stress to health professionals, as it requires the adoption of technology-based measures without the necessary knowledge or adequate training, generating extra pressure on the professional, above and beyond the stress levels experienced by the general public in the face of COVID-19. Parallel to this, the fear of exposure to SARS-CoV-2 at work can cause additional stress and anxiety, in addition to favoring harmful consequences on their psychological health and their performance in the care of patients with cancer [58].

Whether in cancer screening or cancer treatment, it is known that the impact of this pandemic was great. In the context of cancer screening, the impact of COVID-19 on health care systems can be illustrated in terms of loss as it causes loss of life, loss of talent and operational activity, and financial loss. Worldwide, it is estimated that approximately half of elective cancer surgeries have been canceled or postponed during service interruptions caused by the pandemic [58]. This scenario can worsen the cancer statistic in numbers, but still, it is difficult to know how 24–7 care services could impact the health of a patient with cancer even more negatively [58].

The immediate interruptions of cancer therapy generated consequences for the workforce and threats to standard cancer nursing practice. A literature review [59] shows that patients with cancer need specific strategies for cancer management during the current pandemic. In addition, patients with cancer have a higher risk of Sars-CoV-2 infection and unfavorable outcomes when acquiring the virus. The redistribution of health resources can make access to treatments difficult, so the management of patients with cancer must find the balance between benefit and risk [59]. The study also addresses the existence of significant difficulties in the construction of a universal protocol due to the various characteristics of each type of cancer, patients, and oncology services, which means that despite the construction of recommendations and guidelines, there is a need for a case-by-case evaluation.

With regard to palliative care, there are challenges related to the stress and suffering of the moment. In this context, the management of patients with cancer in palliative care questions the premise of generating optimal symptom control and patient comfort. Thus, despite the current scenario, efforts to promote the well-being of the patients must be made [59].

There is still the challenge of assisting patients with cancer in hospitals that are striving to deal with the new coronavirus properly. The risks associated with cancer treatment during the pandemic are not easily resolved. As a result, many patients are not being treated or managed appropriately. The saturation of the health system, which has had resources allocated to COVID-19, threatened the adequate treatment of oncological diseases. Thus, during the ongoing COVID-19 pandemic, it is expected that the mortality of patients with cancer will continue to increase either by the infection or by the failure to provide adequate cancer treatment [59].

The COVID-19 pandemic has presented several challenges for oncology services, and nurses play a key role in the care and management of COVID-19 worldwide. The “Nursing Now” campaign launched in London in February 2018 values the nursing profession, and in 2020, the world celebrated the bicentennial of the birth of Florence Nightingale, the mother of contemporary nursing. Florence Nightingale stood out for her work in the Crimean War, and so many years later, the nursing profession continues to stand out worldwide for being essential on the front line in the fight against the pandemic [57].

### Limitations

The limitations of this study include the scoping literature review method, which may not have apprehended all potentially relevant studies, in addition to the constant appearance of new publications about COVID-19, making it difficult to include all relevant studies. The focus on scientific publications on nursing may also have excluded some relevant studies with implications for the generalization of the conclusions. We adopted the inclusion of a third reviewer as a strategy to strengthen the research design and overcome the limitations.

### Conclusion

The knowledge produced in this research made it possible to identify several strategies used by oncology nurses to face the COVID-19 pandemic in the international scenario. Reports concerning the restructuring of services and the team's reactions to the pandemic predominated. The findings show great appreciation and recognition of nurses' role, especially in managing institutions, restructuring hospital and home care services, and follow-up of patients with cancer during the pandemic.

Difficulties experienced in the daily lives of health professionals and the emotional issues of being a nurse amid the pandemic were discussed, but there was a lack of studies on strategies to support these professionals. Another gap identified was the scarcity of clinical studies on the activities developed by oncology nurses. Therefore, there is a need for clinical research in the oncology area and emotional coping strategies to support oncology nurses.

## Abbreviations

JBI: Joanna Briggs Institute
ONS: Oncology Nursing Society
PPE: personal protective equipment
WHO: World Health Organization
